# Posture and firmness changes in a pressure-relieving air mattress affect cough strength in elderly people with dysphagia

**DOI:** 10.1371/journal.pone.0208895

**Published:** 2018-12-11

**Authors:** Norimichi Kamikawa, Hironobu Hamada, Kiyokazu Sekikawa, Hikaru Yamamoto, Yoshiya Fujika, Teruki Kajiwara, Fumiya Aizawa, Ippo Otoyama

**Affiliations:** 1 Department of Physical Analysis and Therapeutic Sciences, Graduate School of Biomedical and Health Sciences, Hiroshima University, Hiroshima, Japan; 2 Department of Rehabilitation, Faculty of Health Sciences, Hiroshima Cosmopolitan University, Hiroshima, Japan; Berner Fachhochschule, SWITZERLAND

## Abstract

Dysphagia is the major pathophysiologic mechanism leading to aspiration pneumonia in the elderly. Elderly people with dysphagia who show low levels of the cough peak flow (CPF) are at greater risk for aspiration pneumonia. It has been reported that CPF values were significantly lower in the “soft” versus “hard” mode of a pressure-relieving air mattress in healthy volunteers in a supine position. Parameters such as spinal curvature, however, were not evaluated in detail. In this study, we clarified whether the changes in posture associated with two different firmness levels of a pressure-relieving air mattress were associated with cough production and related factors in the elderly with dysphagia. The body sinking distance, pelvic tilt angle, and immersion of the lumbar spine were measured to evaluate changes in posture. Forty subjects met the study criteria for dysphagia. The “soft” mode showed significantly lower CPF values than the “hard” mode (soft 274.9 ± 107.2 L/min vs. hard 325.0 ± 99.5 L/min, MD 50.0 95%CI 33.1–66.9 P < 0.001). Values of forced vital capacity (FVC) and maximal inspiratory pressure (PImax) were significantly lower in the “soft” mode than in the “hard” mode (MD 0.10 95%CI 0.04–0.17, P = 0.002, MD 3.2 95%CI 0.9–5.5, P = 0.007, respectively). Although there was no significant difference between the two firmness levels, maximal expiratory pressure (PEmax) values also tended to be lower in the “soft” than in the “hard” mode, (MD 2.9 95%CI -0.6–6.3 P = 0.1). At both firmness levels, CPF values were significantly correlated with FVC, PImax, and PEmax. The difference in sinking distance in the anterior superior iliac spine was significantly larger than that in the lesser tubercle of the humerus and patella. Additionally, in the soft mode, the pelvic tilt angle and contact area around the lumbar spine were significantly larger than those observed in the “hard” mode. Parameters associated with the production of cough, including inspiratory muscle strength, lung volume, and ultimately CPF, may be affected by immersion of the lumbar spine and curvature of the spine that results from the “soft” mode in elderly patients with dysphagia.

## Introduction

More than 80% of patients with pneumonia aged over 70 years have been reported to suffer from aspiration pneumonia [1]. Aspiration pneumonia has been reported to cause high mortality, especially among the elderly [2]. The risk factors for aspiration pneumonia are sputum suctioning, dysphagia, dehydration, and dementia [3]. Dysphagia is the major pathophysiologic mechanism leading to aspiration pneumonia in the elderly [4]. It has been reported that elderly people with dysphagia who show low levels of cough peak flow (CPF) (an index of voluntary cough strength) are at a greater risk for aspiration pneumonia and respiratory infection [5, 6]. Therefore, it is presumed that maintenance of high levels of CPF in elderly patients with dysphagia in any clinical setting is important.

There are several factors that affect the CPF value, such as thorax extensibility, lung volume, respiratory muscle strength [7–14], posture [15, 16], and the firmness levels of the pressure-relieving air mattress [17]. We previously reported that CPF values were significantly lower in the “soft” mode than those in the “hard” mode of pressure-relieving air mattresses in healthy volunteers in a supine position [17]. However, the mechanism of change in posture between the different levels of firmness has not been well defined.

In this study, we evaluated the changes in posture in two levels of firmness of a pressure-relieving air mattress to determine the parameters that affected cough production in elderly people with dysphagia.

## Materials and methods

### Participants

Among the 81 community-dwelling elderly people aged 65 years or older who participate in the care prevention service hosted by the community, 40 people with dysphagia were enrolled. Dysphagia was defined by scores of 3 or higher on the 10-item Eating Assessment Tool (EAT-10), as previously reported [18]. The ethics committee of the Hiroshima University Graduate School of Health Sciences approved this study (#1602). All participants provided written informed consent.

### Assessments: Changes in body position and settings for the pressure-relieving air mattress

The participants were in the supine position on a pressure-relieving air mattress (GRANDE; Molten, Co., Ltd, Tokyo, Japan) for all assessments. To eliminate the bed angle and the effect of the pillow, the supine position was at 0° inclination, and the position of the head was flat with no pillow. The air mattress settings and the body positions used for the assessments were consistent with the previous reports [17]. There are 4 firmness levels for the air mattress, ranging from softest (“soft”) to hardest (“hard”) levels that were used in this study. The inner pressure levels of the air mattress were measured using a digital pressure gauge (PGI; Molten Co., Ltd, Hiroshima, Japan) for confirmation, as previously reported [17].

### Measurement of cough strength and lung volume

CPF and forced vital capacity (FVC), an indicator of lung volume, were measured using an electronic spirometer (Autospiro AS-507, Minato Medical Science. Co., Ltd Osaka, Japan). FVC was measured as per the standard methods of the American Thoracic Society [19]. Three measurements for CPF and FVC were made by the examiner using the sensor. Three practices were attempted before measuring the CPF. The statistical analysis used the highest values for CPF and FVC obtained at each firmness level.

### Respiratory muscle strength measurements

Respiratory muscle strength was assessed as the maximal inspiratory pressure (PImax) and maximal expiratory pressure (PEmax) using a measurement device (AAM377, Minato Medical Science. Co., Ltd, Osaka, Japan) connected with an electronic spirometer (Autospiro AS-507), as previously reported [17]. After 3 practice measurements, PImax and PEmax values were evaluated three times at each air mattress firmness level, and the highest values were included in the statistical analysis.

### Body sinking distance measurement

The amount that the body sank into the air mattress was quantified by measuring, without any activity, three points on the body: left anterior superior iliac spine (ASIS), the left lesser tubercle of the humerus, and the left patella.

Each bony protuberance was labelled with a marker, and the distance from the marker to a reference line installed at 1000 mm above the floor was measured with the air mattress in both “soft” and “hard” modes, using a two-dimensional motion analysis system (ICpro-2DdAF for Windows, Hu-Tech, Tokyo, Japan). The following formula was used to calculate the difference in the sinking distances of the “soft” and “hard” modes:

Difference in sinking distance = sinking distance in “soft” mode–sinking distance in “hard” mode.

### Measurement of pelvic tilt angle

**Fig 1:**
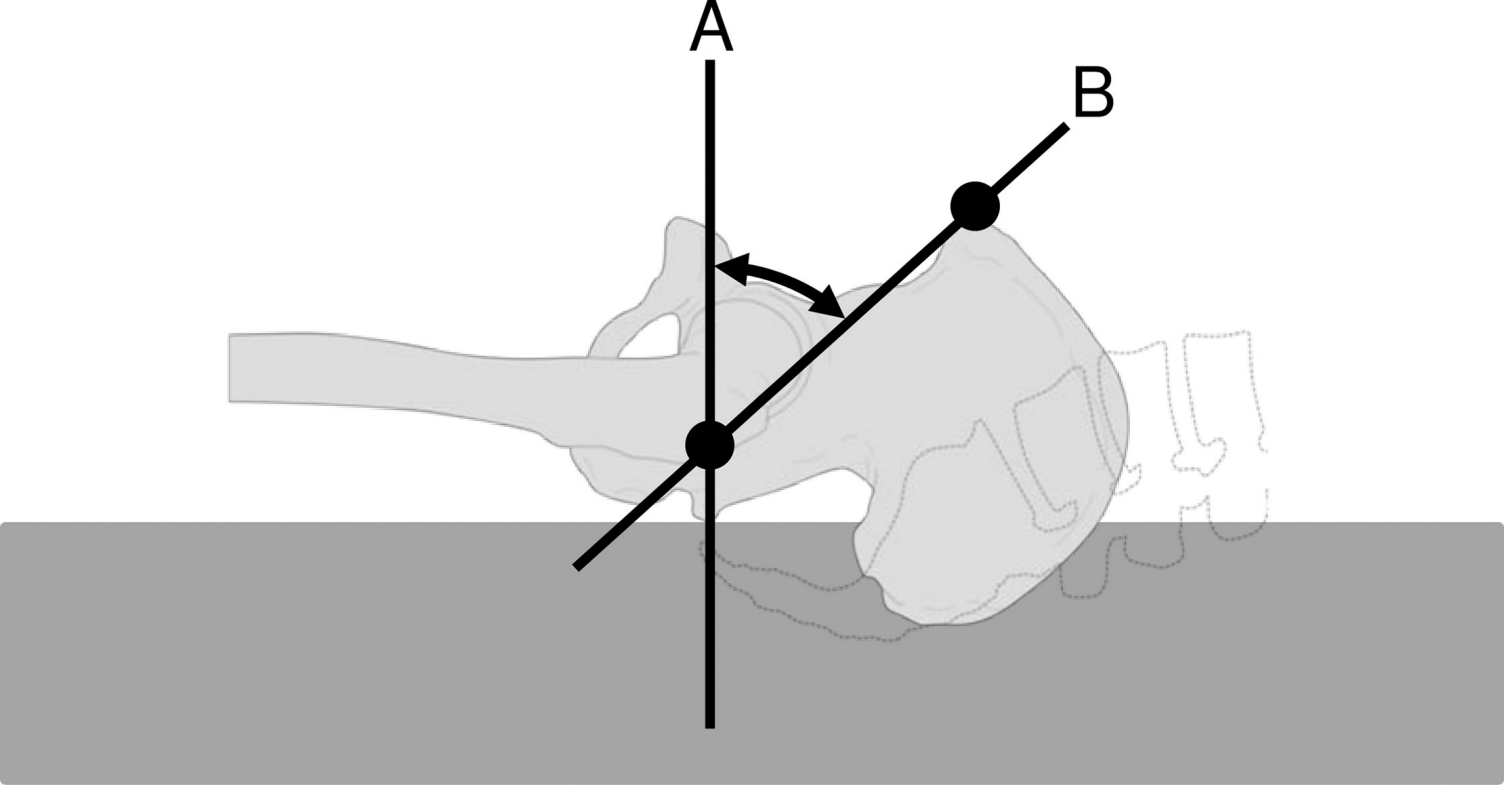
Pelvic tilt angle. (A) Vertical line. (B) The line connecting the ASIS and greater trochanter.

The pelvic tilt angle, which is the angle between vertical line and the line connecting the ASIS and greater trochanter, was measured (Fig 1). The angle increases when the pelvis tilts posteriorly and decreases when the pelvis tilts anteriorly.

At each bony protuberance, a marker was placed, and the angle between the lines was measured in both the “soft” and “hard” modes of the air mattress, using a two-dimensional motion analysis system (ICpro-2DdAF for Windows).

### Measurement of immersion of the lumbar spine

Previous studies have reported that a soft mattress leads to deeper immersion of the buttocks and lumbar spine with a greater spinal curvature [20] and increases the contact area between the mattress and body [21]. Therefore, we measured the contact area around the lumbar spine as an indicator of immersion of the lumbar region.

The tactile sensor sheet of the pressure-distribution measurement system (Clinseat BIG-MAT, NITTA. Co., Ltd Osaka, Japan) was placed on the air mattress. The contact area from the first lumbar vertebra to the posterior superior iliac spine (PSIS) on the body was measured in both the “soft” and “hard” modes of the air mattress.

### Statistical analysis

Results are expressed as means ± standard deviation (SD). A one-way analysis of variance and Tukey HSD test were used to compare the differences in sinking distances measured at three body points. The comparison of the inner mattress pressure, CPF, FVC, PImax, PEmax, pelvic tilt angle, and contact area around the lumbar spine was determined at the two firmness levels using a paired *t* test. The mean difference (MD) and 95% confidence intervals (95%CI) were calculated for continuous outcomes. Statistical analysis was performed using SPSS software ver. 21 for Windows (IBM Japan, Tokyo, Japan). *P* values less than 0.05 were considered statistically significant.

## Results

Eighty-one subjects were screened, and 40 met the study criteria for dysphagia. Among the 40 participants, the characteristics and spirometric parameters were as follows: age, 75.4 ± 4.1 years; height, 157.7 ± 8.2 cm; weight, 57.1 ± 8.8 kg; body mass index, 22.9 ± 2.9 kg/m^2^; percent body fat, 32.1 ± 6.3%; EAT-10 score, 4.0 ± 1.1; percent of predicted FVC, 85 ± 15%; and percent of predicted forced expiratory volume in 1 s, 89 ± 16%.

**Fig 2:**
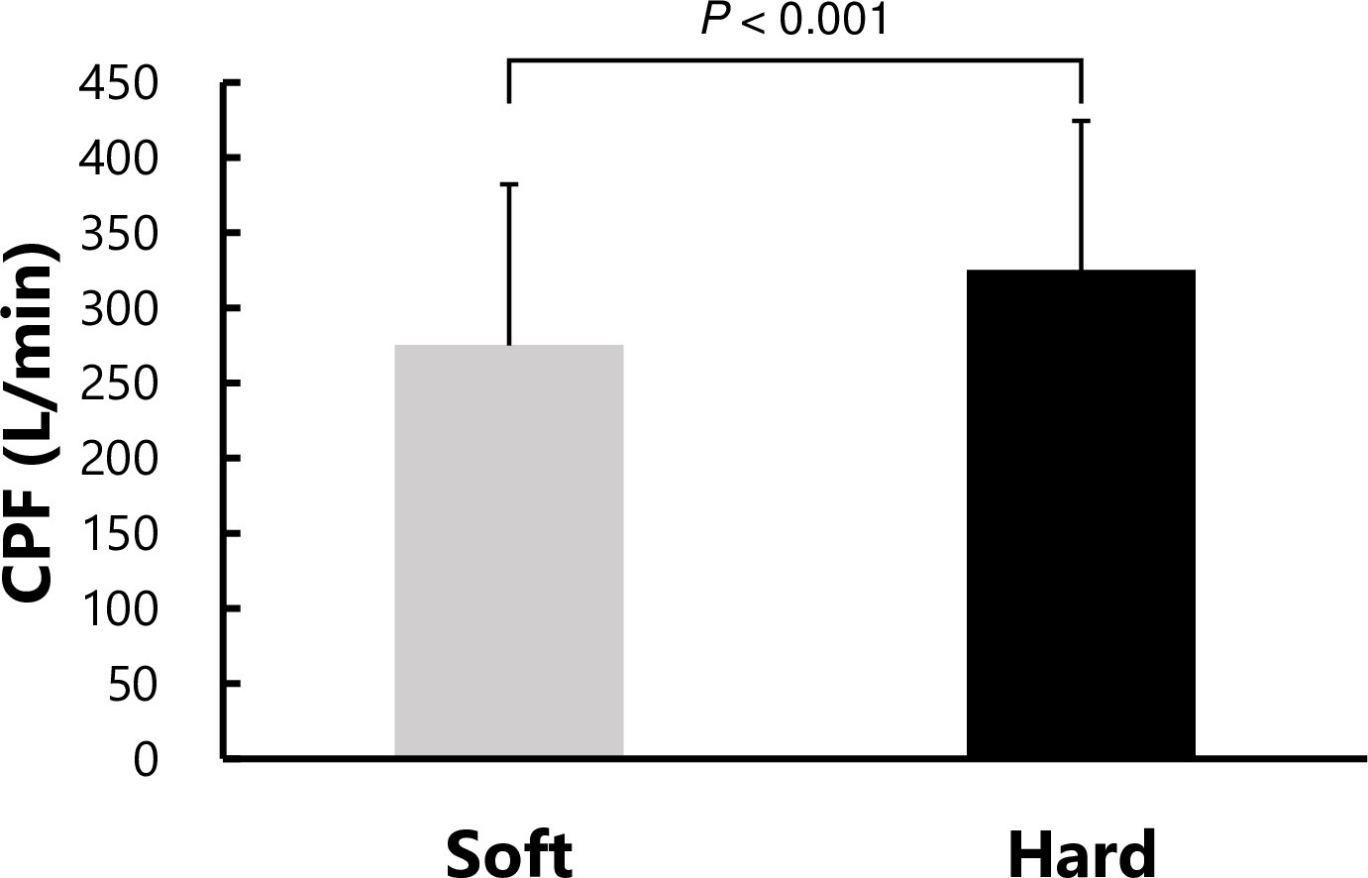
CPF at two different firmness levels. CPF = cough peak flow. Values are expressed as mean ± standard deviation (SD).

The inner pressure of the air mattress was significantly lower in the “soft” than in the “hard” mode (soft 23.4 ± 2.6 hPa vs. hard 33.4 ± 2.6 hPa, *P* < 0.001). CPF was significantly lower in the “soft” mode than in the “hard” mode (soft 274.9 ± 107.2 L/min vs. hard 325.0 ± 99.5 L/min, MD 50.0 95%CI 33.1–66.9 *P* < 0.001), as shown in Fig 2. FVC and PImax were significantly lower in the “soft” mode compared with the “hard” mode (MD 0.10 95%CI 0.04–0.17 *P* = 0.002, MD 3.2 95%CI 0.9–5.5 *P* = 0.007, respectively), as summarized in Table 1. PEmax values also tended to be lower in “soft” rather than in the “hard” mode, although there was no significant difference between the two firmness levels (MD 2.9 95%CI -0.6–6.3 *P* = 0.1).

**Table 1:** Cough parameters at two different firmness levels.

	Soft	Hard	*P* value	MD (95%CI)
FVC (L)	2.21 ± 0.65	2.31 ± 0.59	0.002	0.10 (0.04 –0.17)
PImax (cmH_2_O)	41.5 ± 19.6	44.7 ± 17.5	0.007	3.2 (0.9 –5.5)
PEmax (cmH_2_O)	52.2 ± 17.6	55.1 ± 17.8	0.1	2.9 (-0.6 –6.3)

FVC = forced vital capacity, PImax = maximal inspiratory pressure, PEmax = maximal expiratory pressure.

Values are expressed as mean ± standard deviation (SD).

**Fig 3:**
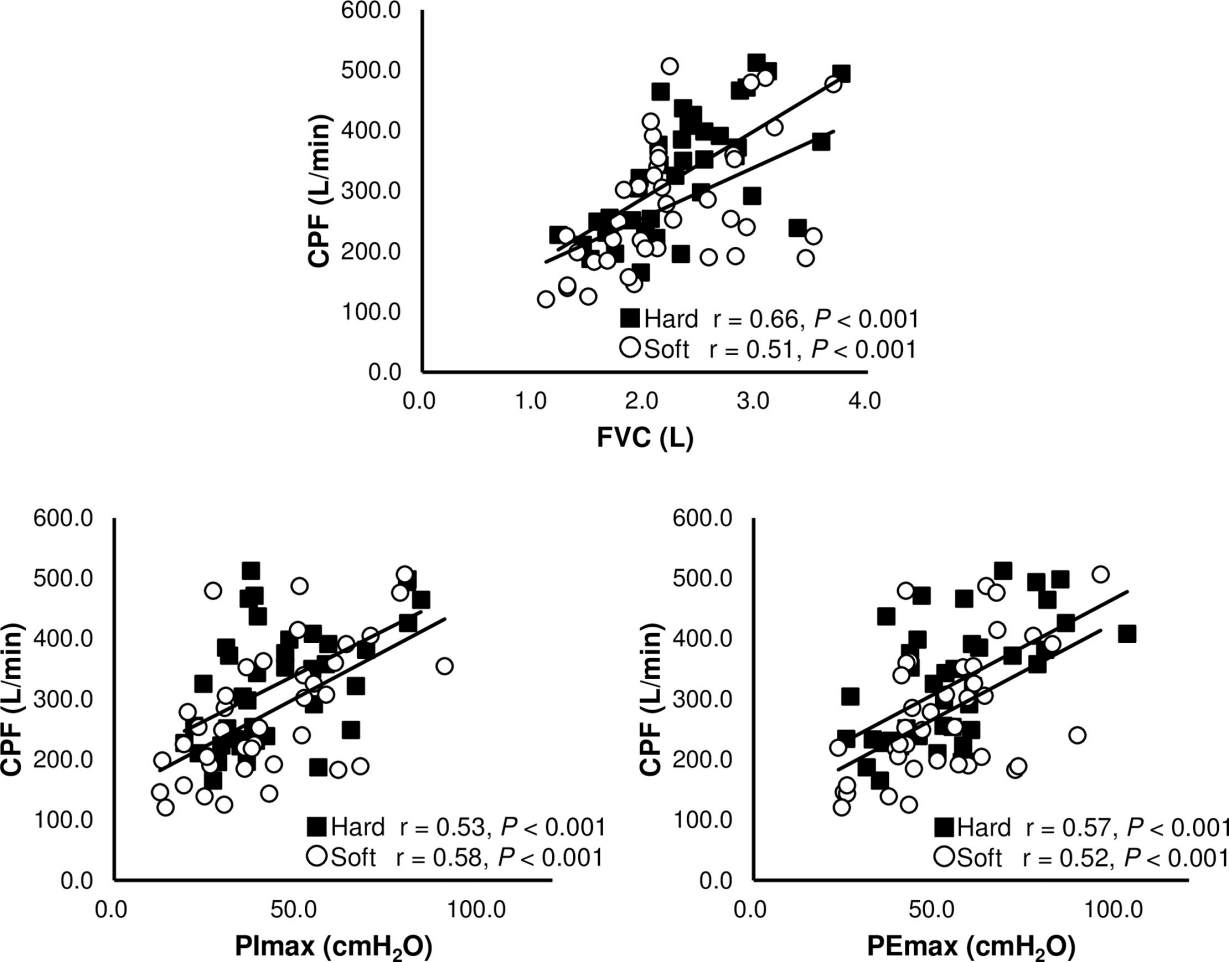
Correlation between parameters associated with cough production and CPF at two different firmness levels. CPF = cough peak flow, FVC = forced vital capacity, PImax = maximal inspiratory pressure, PEmax = maximal expiratory pressure, r = Pearson correlation coefficient.

Fig 3 shows the correlation between the parameters associated with the production of cough and CPF at the two different firmness levels. CPF was significantly correlated with FVC, PImax, and PEmax at both firmness levels (Fig 3).

The differences between the “soft” and “hard” modes for the sinking distances measured at the lesser tubercle of the humerus, patella, and ASIS were -2.5 ± 1.7, -1.7 ± 1.2, and -3.7 ± 2.0 respectively. The difference in sinking distance at the ASIS was significantly greater than those of the lesser tubercle of the humerus (*P* < 0.01) and the patella (*P* < 0.001). The pelvic tilt angle was significantly larger in “soft” mode (38.2 ± 7.6°) than that in “hard” mode (34.7 ± 7.6°, *P* < 0.001). The contact area around the lumbar spine was significantly greater in “soft” mode (418.8 ± 54.7 cm^2^) than that in “hard” mode (363.2 ± 59.5 cm^2^, *P* < 0.001).

## Discussion

In this study, we evaluated whether changes in posture associated with two firmness levels of a pressure-relieving air mattress were associated with changes in CPF values in the elderly people with dysphagia. In the “soft” mode, the CPF, FVC, and PImax values were significantly lower than those in the “hard” mode and were comparable to our previous study results [17]. The CPF values were significantly correlated with FVC, PImax, and PEmax at both firmness levels. The pelvic tilt angle and contact area around the lumbar spine were significantly greater in the “soft” mode than those in the “hard” mode. These findings suggest that CPF, respiratory muscle strength, and lung volume might be affected by changes in posture at different firmness levels. The decline in CPF in the elderly people with dysphagia increases the risk of aspiration pneumonia and respiratory infection [5, 6]. Bianchi et al., reported that the cough of dysphagic patients with pulmonary complications is significantly reduced, and the receiver operation characteristic curve analysis shows that CPF levels lower than 242 L/min predict the occurrence of pulmonary complications [5]. In this study, 7 subjects showed CPF levels less than 242 L/min in soft mode of pressure-relieving air mattresses. However, the levels were increased to more than 242 L/min in the hard mode. Our study suggested that it may be important to use firm mattresses in this population.

The present study demonstrated that cough strength in the elderly with dysphagia was affected by the two firmness levels of a pressure-relieving air mattress, which was comparable to the results of our previous study in healthy young men [17]. In that study, we evaluated the difference between the sinking distances of the two firmness levels in order to quantify how much the body sank into the air mattress. However, we were not able to determine details regarding posture changes (such as spinal curvature). In the present study, we measured two parameters such as pelvic tilt angle and the contact area around the lumbar as well as sinking distances to evaluate the changes of posture between the “soft” and “hard” modes. The pelvic tilt angle and lumbar contact area values in the “soft” mode were significantly larger than those in the “hard” mode. It was considered that immersions of the lumbar area in the “soft” mode was larger than those in the “hard” mode.

It has been reported that posterior pelvic tilt decreases lumbar lordosis [22, 23], and that a soft mattress increases the lumbar contact area [21, 24] and decreases lumbar lordosis [21]. These studies suggested that deeper immersion with spinal curvature decreased lumbar lordosis, which in turn was associated with pulmonary function. It was reported that spinal curvature reduces the motion of the ribs and the total lung capacity [25]. Fang et al., reported that a posture that reduces lumbar lordosis may compress organs and impede diaphragm movement, leading to a decrease in FVC [16]. Furthermore, adjustments to spinal alignment have been reported to change the volume of air available to the lung and/or influence the efficacy of contraction of the diaphragm and other respiratory muscles [16]. FVC and PImax values were significantly lower for the “soft” mode than those for the “hard” mode in this study, and PEmax values also tended to be lower. Our study suggests that changes in posture with immersion of the lumbar spine and spinal curvature accompanying posterior pelvic tilt may be associated with the CPF of elderly people with dysphagia.

### Limitations

The first limitation of this study is its small sample size. Second, the participants were not patients with aspiration pneumonia. Further studies are needed to clarify the effects of different firmness levels in pressure-relieving air mattresses on the cough strength of patients with aspiration pneumonia. Continuing studies would provide effective information regarding the environmental factors for patients with aspiration pneumonia and those undergoing pulmonary rehabilitation.

## Conclusions

The “soft” mode of a pressure-relieving air mattress caused posterior pelvic tilt and lumbar lordosis, decreased lung volume, respiratory muscle strength, and CPF in the supine position. This study suggests that if a patient is on a pressure-relieving mattress and is struggling to cough effectively, the clinician could consider changing the mattress from soft to hard mode to help the patient generate a stronger cough.

## Supporting information

S1 File Data used for analyses of the study. (XLSX)

S1 Fig Supporting information for Fig 2. (PDF)
