# Supplementary figures and images for: Posture and firmness changes in a pressure-relieving air mattress affect cough strength in elderly people with dysphagia
Source: PLoS One. 2018 Dec 11;13(12):e0208895. doi: 10.1371/journal.pone.0208895 (PMC6289577; doi:10.1371/journal.pone.0208895)

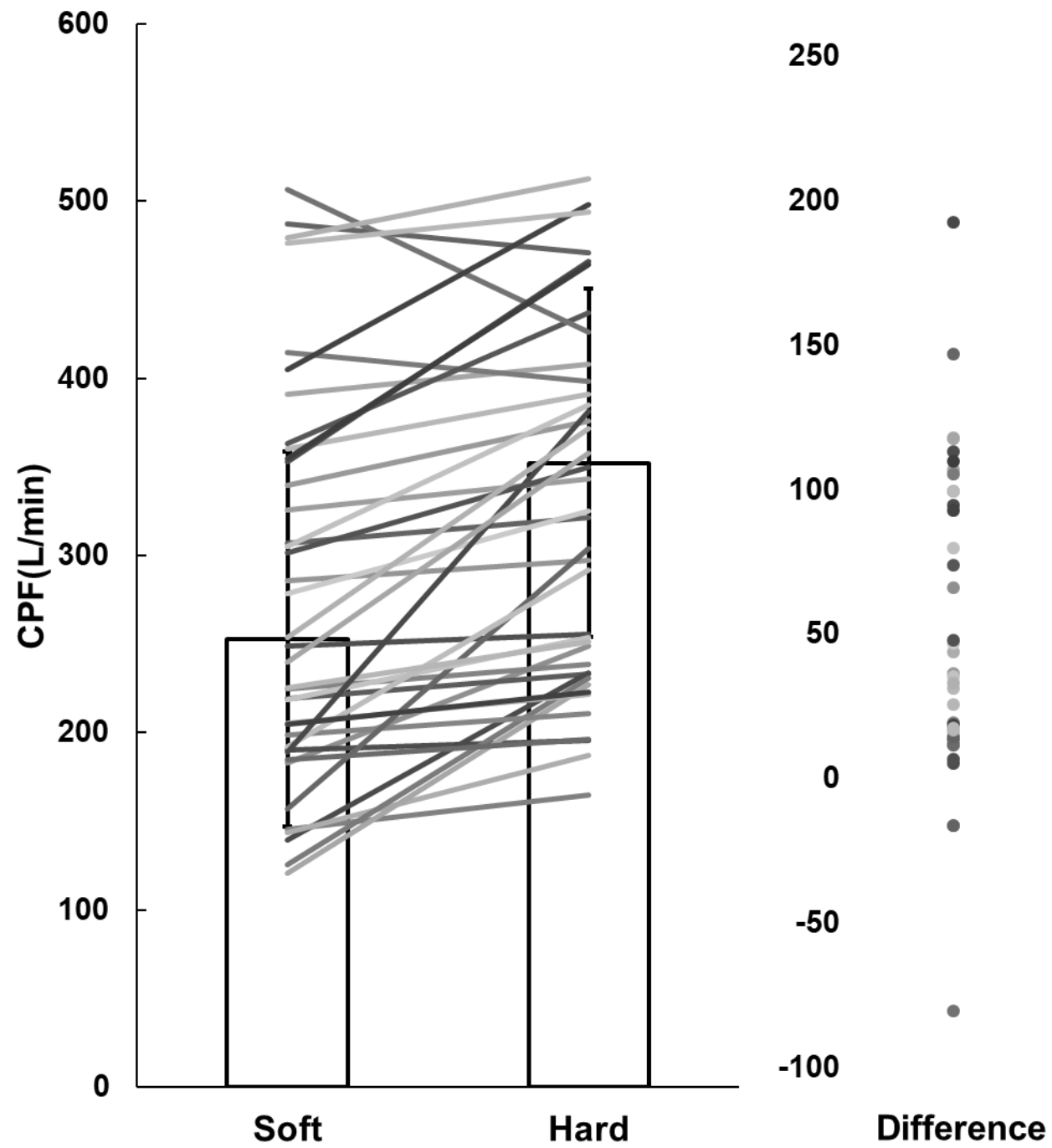

Supplement: S1 Fig — (PDF) [file pone.0208895.s002.pdf]
